# Impact of extramedullary disease in AML patients undergoing sequential RIC for HLA-matched transplantation: occurrence, risk factors, relapse patterns, and outcome

**DOI:** 10.1007/s00277-023-05281-8

**Published:** 2023-06-10

**Authors:** Alessia Fraccaroli, Daniela Vogt, Margarete Rothmayer, Karsten Spiekermann, Friederike Pastore, Johanna Tischer

**Affiliations:** Department of Internal Medicine III, University Hospital Munich, Ludwig-Maximilian-University (LMU), Munich, Germany

**Keywords:** AML, EMD and relapse, Risk factors, Allogeneic HSCT, Sequential RIC

## Abstract

We sought to evaluate the role of extramedullary disease (EMD) in sequential RIC retrospectively analyzing data of 144 high-risk AML patients undergoing HLA-matched transplantation. Median long-term follow-up was 11.6 years. Eighteen percent of patients (*n* = 26/144) presented with extramedullary AML (EM AML) or a history of EMD at time of transplantation. Overall relapse rate was 25% (*n* = 36/144) with 15% (*n* = 21/144) of all patients developing isolated BM relapse and 10% (*n* = 15/144) developing EM AML relapse with or without concomitant BM relapse (EM ± BM). Manifestation of EM relapse after transplantation occurred frequently at multiple sites and presented mostly as solid tumor mass. Only 3/15 patients with EM ± BM relapse showed a prior EMD manifestation. EMD prior to allogeneic transplantation had no impact on post-transplant OS when compared to non-EMD (median post-transplant OS 3.8 years versus 4.8 years; ns). Risk factors (*p* =  < 0.1) for EM ± BM relapse included younger age and a higher number of prior intensive chemotherapies, whereas the presence of chronic GVHD was a protective factor. Median post-transplant OS (15.5 months vs. 15.5 months), RFS (9.6 months vs 7.3 months), and post-relapse OS (6.7 months vs. 6.3 months) were not significantly different between patients with isolated BM vs. EM ± BM relapse. Taken together, occurrence of EMD prior to as well as of EM ± BM AML relapse after transplantation was moderate, presenting mostly as solid tumor mass after transplantation. However, diagnosis of those does not seem to influence outcomes after sequential RIC. A higher number of chemotherapy cycles prior to transplantation was identified as recent risk factor for EM ± BM relapse.

**Supplementary information:**

The online version contains supplementary material available at 10.1007/s00277-023-05281-8.

## Introduction

Allogeneic hematopoietic stem cell transplantation (allo-HSCT) is a potential curative treatment option for patients with acute myeloid leukemia (AML) [1]. Despite the reduction of treatment-related mortality by the introduction of reduced-intensity conditioning (RIC) regimen and improvements in supportive treatments [2], relapse after allogeneic transplantation remains the most common cause that negatively affects long-term outcome [3], especially in patients suffering from high risk AML [4]. Whereas conventional conditioning regimens led to unsatisfying results in this patient cohort, sequential conditioning protocols, such as FLAMSA-RIC, have been successfully established [5, 6] enabling transplantation with acceptable toxicity profile and relapse rates. Thus, they advanced to standard of care at our and other institutions in high-risk, relapsed and refractory AML. Despite various analyses describing the feasibility and applicability throughout different donor transplantation settings [5, 7–10] and underlying diseases [11, 12], up to now, there is no study focusing on relapse pattern within this widespread used conceptual platform.

Relapse of AML after allo-HSCT can occur either as isolated relapse within the bone marrow (BM), or as extramedullary (EM) relapse either isolated or more frequently combined with concurrent relapse in the BM. EM AML relapse after allo-HSCT has been described with frequencies between 1 and 15% [13, 22] and up to 32% after haploidentical HSCT [23]. It remains controversial whether EM relapses after allogeneic HSCT yield a better [13, 15, 17] or a similar [16, 23] prognosis to relapses in the BM, mostly due to limitations caused by small patient numbers. Furthermore, little data is known regarding the features of AML with extramedullary disease (EMD), its relapse patterns and risk factors, as well as its impact on outcome when diagnosed prior to or after transplantation in a sequential RIC setting [5, 6, 10, 24].

The aim of our study was to assess the occurrence, the prognostic impact and risk factors for EM AML relapse development following a sequential-RIC allo-HSCT. Furthermore, we aimed to elucidate if differences in relapse disease presentation (BM relapse versus EMD with or without BM relapse) caused differences in outcomes, with a special focus on the role of a prior extramedullary manifestation and extramedullary relapse patterns.

## Patients and methods

### Patients

Analyses are based on patients with AML that underwent an HLA-matched allogeneic RIC-HSCT in our center between 2006 and 2010. All patients received sequential treatment based on FLAMSA (fludarabin (4 × 30 mg/m^2^), amasacrin (4 × 100 mg/m^2^), cytarabine (4 × 2000 mg/m^2^), followed by RIC composed of either total body irradiation (TBI) with 400 cGy or busulfan (Bu) (8 × 8 mg/kg). All patients received a GvHD prophylaxis with anti-thymocyte globulin (ATG, 3 × 10 mg/kg body weight or 3 × 20 mg/kg body weight in case of unrelated donors) plus cyclophosphamide (Cy) (2 × 40 mg/kg and 2 × 60 mg/kg in case of unrelated donors) followed by post-grafting immunosuppression with either cyclosporine A (CsA)/mycophenolate mofetil (MMF), tacrolimus/MMF or sirolimus/MMF or methotrexate (MTX).

### Cytogenetic and molecular diagnostics

Cytogenetic and molecular analyses were performed in the leukemia diagnostic laboratory, following standard guidelines. For cytogenetic analyses ≥ 20 metaphases were assessed. Cytogenetic risk was defined according to the Medical Research Council (MRC) classification [25]. Mutations of *NPM1*, *FLT3*-ITD, *FLT3*-TKD and *KMT2A*-PTD (*MLL*-PTD) were assessed as previously published [26, 27].

### Statistical methods

In our assessment for their potential prognostic impact on relapse risk, we included clinical parameters, disease parameters, therapeutic risk factors as well as post-transplant characteristics.

The following clinical and disease biology related variables were assessed: recipient age at time of allogeneic HSCT gender, ECOG status at allogeneic HSCT (0/1 versus (vs) 2), presence of prior EM manifestation, HCT-CI score (< 3 vs ≥ 3) [28], WBC at diagnosis, BM blasts at diagnosis, peripheral blasts at diagnosis, de novo vs. non de novo AML, FAB type, cytogenetic risk according to MRC, CN-AML vs. non-CN-AML, mutation status of *NPM1*, *FLT3*-ITD, *FLT3*-TKD, *KMT2A*-PTD, expression of T-cell markers, expression of CD56. Transplant-related variable included prior number of intensive chemotherapy cycles (continuous as well as < 2 vs ≥ 2), d16 blast clearance during induction treatment (< 10% vs ≥ 10%) [29], time from diagnosis to allogeneic HSCT, remission status before allogeneic HSCT (CR/CRi) vs. relapse/refractory disease vs. upfront allogeneic HSCT, donor characteristics such as HLA compatibility, donor type (related identical vs. unrelated identical vs. unrelated different), gender of donor/recipient, CMV state of donor/recipient, stem cell source (peripheral blood (PB) vs. BM), stem cell dose (CD34 + cells/kg), conditioning regimen and use of ATG 10 mg vs. 20 mg, GVHD prophylaxis (CsA/ MMF vs. others). Post-transplant characteristics that were assessed included: time from transplant until engraftment, time from transplant to relapse, time from transplant to onset of acute GVHD (aGVHD), acute and chronic GVHD (cGVHD) including severity grades as well as localization of aGVHD and cGVHD.

Statistical comparisons of risk factors between the types of relapses were assessed using the chi-square or Fisher exact test for categorical variables and the Kruskal–Wallis and Mann–Whitney *U* test for continuous factors. A binary logistic regression was performed to identify risk factors for dichotomous outcome parameters (EM relapse vs no EM relapse, BM relapse vs no BM relapse).

Post-transplant overall survival (OS) was defined as interval from date of allogeneic HSCT until death or last follow-up. Relapse free survival (RFS) was defined as time from allogeneic HSCT until relapse or last follow-up. Post-relapse overall survival was defined as interval from relapse after allogeneic HSCT and until death or last follow-up. Median follow-up time for survivors was calculated by the reverse Kaplan Meier method. Probabilities of post-transplant OS, RFS and post-relapse OS were calculated using the Kaplan–Meier method.

## Results

### Patient, disease, and transplant characteristics

Patient-, disease- and transplant characteristics are summarized in Table 1. Between 2006 and 2010, 144 adult patients with AML were treated with FLAMSA-RIC using TBI/Cy or FLAMSA-RIC using Bu/Cy followed by allogeneic HSCT at our center. Median age at allo-HSCT was 49 years (range: 18–71 years), 49% were female and 85% showed an ECOG of 0 or 1 at transplant (Table 1). 70% of patients had de novo AML, 19% secondary AML (sAML) and 10% therapy-related AML (tAML). The majority of patients had diagnosis of AML with an intermediate and adverse cytogenetic risk (68% and 24%, respectively). 52% were cytogenetically normal AML with mutations of *NPM1*, *FLT3*-ITD and *FLT3*-TKD in 35%, 27%, and 9% of cases. Most patients were transplanted in relapse or with refractory disease (51%).

**Table 1:** Clinical, disease and transplant characteristics in all patients and patients with prior EM disease versus non-EM disease

	All (*n* = 144)		Non-EM AML (*n* = 118)		EM AML (*n* = 26)		*P* (Non-EM AML vs EM AML)
	*N* (%)	Median (range)	*N* (%)	Median (range)	*N* (%)	Median (range)	
Clinical characteristics							
Total patients	144 (100)		118		26		
Age at allogeneic HSCT, years		49 (18–71)		49 (19–71)		54 (18–69)	0.891
Female	71 (49)		53 (45)		18 (69)		**0.025**
ECOG at allo HSCT							0.650
ECOG 0/1	122 (85)		99 (84)		23 (88)		
ECOG 2	22 (15)		19 (16)		3 (12)		
Prior EM manifestation at diagnosis	10 (7)		0 (0)		16 (62)		**–**
Prior EM manifestation before allo HSCT	26 (18)		0 (0)		26 (100)		**–**
HCT-CI score, ≥ 3	30 (21)		26 (22)		4 (15)		0.420
WBC at diagnosis, × 10^9^/L		8.2 (0.2–255)		5.4 (0.2–255)		30 (1–195)	**0.030**
Bone marrow blasts at diagnosis, %		60 (4–98)		57 (4–98)		75 (4–90)	0.125
Peripheral blasts at diagnosis, %		29 (0–96)		27 (0–96)		34 (14–90)	0.136
Disease biology							
De novo AML	101 (71)		80 (68)		21 (81)		0.191
FAB type							0.348
M0	7 (6)		7 (6)		0 (0)		
M1	26 (23)		21 (18)		7 (27)		
M2	44 (36)		38 (32)		6 (23)		
M4	30 (24)		21 (18)		9 (35)		
M5	10 (8)		7 (6)		3 (12)		
M6	3 (2)		3 (3)		0 (0)		
M7	1 (0.8)		1 (1)		0 (0)		
Cytogenetic risk group (MRC)							0.498
favorable	12 (8)		9 (8)		3 (12)		
intermediate	94 (68)		75 (64)		19 (73)		
adverse	33 (24)		29 (25)		4 (15)		
CN-AML	73 (52)		59 (50)		14 (54)		0.815
*NPM1* mutated	38 (35)		28 (24)		10 (38)		0.068
*FLT3*-ITD	31 (27)		23 (19)		8 (31)		0.156
*FLT3*-TKD mutated	6 (10)		3 (3)		3 (12)		0.058
*MLL*-PTD	10 (10)		9 (8)		1 (4)		1.000
Expression of T cell markers before allo HSCT	56 (48)		22 (19)		4 (15)		1.000
Expression of CD56 before allo HSCT	21 (18)		18 (15)		3 (12)		0.760
Therapy prior to allogeneic HSCT							
Prior intensive chemotherapy cycles, number		2 (1–4)		2 (1–4)		2 (1–3)	0.889
d16 Blast clearance < 10%	94 (72)		75 (64)		19 (73)		0.922
Remission status before allogeneic HSCT							0.064
CR/CRi	56 (39)		44 (37)		12 (46)		
Relapse/Refractory	74 (51)		60 (51)		14 (54)		
Upfront allogeneic HSCT	14 (10)		14 (12)		0 (0)		
Time from diagnosis to allogeneic HSCT (mo)		5.7 (1–56)		4.9 (1.1–56)		7.4 (2.5–25.5)	0.060
Allogeneic HSCT							
Donor type							0.248
Matched related (10/10)	47 (33)		42 (36)		5 (19)		
Matched unrelated (10/10)	77 (53)		61 (52)		16 (62)		
Mismatched unrelated (9/10)	20 (14)		15 (13)		5 (19)		
Donor/recipient gender mismatch	63 (44)		46 (39)		17 (65)		**0.028**
Female donor/Male recipient	23 (16)		18 (15)		5 (19)		
Male donor/Female recipient	40 (28)		28 (24)		12 (46)		
CMV Status, donor/recipient							0.774
Negative/negative	47 (33)		37 (31)		10 (38)		
Negative/positive	13 (9)		10 (8)		3 (12)		
Positive/negative	34 (24)		28 (24)		6 (23)		
Positive/positive	50 (35)		43 (36)		7 (27)		
Graft source							1.000
Peripheral blood stem cells	141 (98)		115 (97)		26 (100)		
Bone marrow	3 (2)		3 (3)		0 (0)		
Stem cell dose, CD34 + cells/kg BW		8.2 (2.7–21.7)		8.1 (2.7–22)		7.9 (3.2–18.8)	0.770
Conditioning regimen							0.799
TBI based	90 (63)		75 (64)		15 (58)		
Drug based	54 (38)		44 (37)		10 (38)		
Post-grafting GvHD prophylaxis							0.079
CsA/MMF	113 (81)		97 (82)		16 (62)		
Others	26 (19)		18 (15)		8 (31)		

Bold typing indicates statistical significance (*p* < 0.05)

*Allo*, allogeneic; *BM*, bone marrow; *EM*, extramedullary; *ECOG*, Eastern cooperative oncology group; *HSCT*, hematopoietic stem cell transplantation; *HCT-CI*, hematopoietic cell transplantation-specific Comorbidity Index; *WBC*, white blood cells; *FAB*, French American British classification; *MRC*, Medical Research Council; *CN-AML*, cytogenetically normal acute myeloid leukemia; *NPM1*, Nucleophosmin1; *FLT3-ITD*, FMS-like tyrosine kinase 3 – internal tandem duplication; *FLT3-TKD*, FMS-like tyrosine kinase 3—tyrosine kinase domain; *MLL-PTD*, mixed lineage leukemia-partial tandem duplication; *CD*, cluster of differentiation; *d*, day; *CR*, complete remission; *CRi*, complete remission with incomplete count recovery; *mo*, months; *CMV*, cytomegalovirus; *kg*, kilogram; *BW*, body weight; *TBI*, total body irradiation; *GvHD*, graft-versus-host disease; *CsA*, ciclosporin A; *MMF*, Mycophenolat-Mofetil; *n*, number

At time of initial AML diagnosis 10 patients (7%) presented with EM manifestation while 16 patients (11%) developed EMD in the course of relapsed or refractory disease, summing up to patients presenting with or with a history of EMD at time of allo-HSCT.

The 26 patients with prior EMD at time of allo-HSCT (EM AML patients) showed a significantly higher WBC at first diagnosis (*p* = 0.03), were mostly female (*p* = 0.025) and were more frequently transplanted with a donor/recipient gender mismatch compared to patients without EM manifestation (*p* = 0.028) compared to 118 patients without EMD before allo-HSCT (Non-EM AML patients) (Table 1). In addition, we observed a trend towards more frequent occurrence of *NPM1* (*p* = 0.068) and *FLT3*-TKD (*p* = 0.058) mutations in patients with EM AML.

Patients had received a median of 2 intensive chemotherapy cycles prior to allo-HSCT regardless of whether EMD manifestation was present or not (*p* = 0.889). Upfront HSCT was only performed in patients without EMD manifestation. Median time from diagnosis to allo-HSCT was 5.7 months in the overall cohort, 4.9 months in the non-EM AML and 7.4 months in EM AML patients (*p* = 0.060). We observed no significant differences in terms of donor type, stem cell source, conditioning regimen and post-grafting immunosuppression (Table 1).

### Outcome: survival and relapse comparing EM AML vs non-EM AML patients

**Fig. 1:**
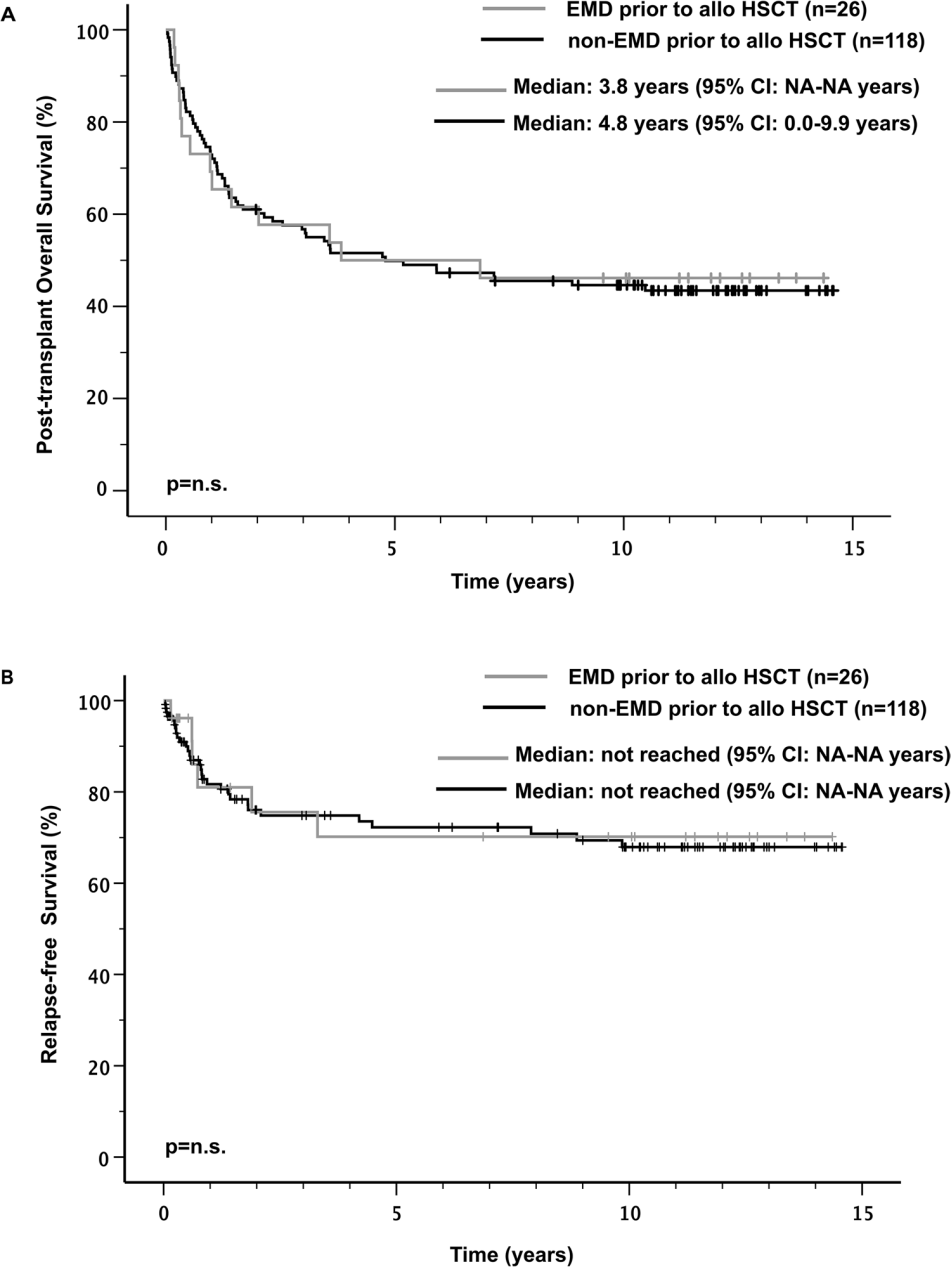
Post-transplant OS (**A**) and RFS (**B**) in patients with or without EMD prior to allo HSCT

Median follow-up for 64 survivors was 11.6 years (95% CI: 10.9–12.3 years). Median post-transplant OS for all 144 patients was 4.8 years (95% CI: 0.3–9.3) with a probability of OS at 3 years post-transplant of 57% (Figure S1A). EMD prior to allo-HSCT had no impact on post-transplant OS when compared with the non-EMD group (median post-transplant OS 3.8 years [95% CI: NA] versus 4.8 years [95% CI: 0.0–9.9 years], respectively and OS at 3 years 58% versus 57%, respectively) (Fig. 1A).

Median RFS was not reached (Figure S1B) and no statistical difference could be detected between patients with EMD at time of allo-HSCT and those without EMD (Fig. 1B). RFS at 3 years were 76% and 75%, respectively.

Cumulative relapse rates for the entire cohort at 3, 5, and 10 years after allogeneic HSCT were 20%, 23%, and 25%, respectively (data not shown). Relapse rate after allo-HSCT did not differ between patients with non-EM AML (*n* = 30/118, 25%) and patients that were diagnosed with prior EM AML (*n* = 6/26, 23%) (*p* = n.s.).

### Relapse types and patterns

Thirty-six patients (25%) relapsed after allo-HSCT; fifteen of these showed EM relapse, either as isolated EM manifestation (*n* = 4) or concurrent to a BM relapse (*n* = 11), hereafter referred to as EM ± BM relapse.

One of 36 patients that relapsed was alive at last follow-up, 89% had died from leukemia (*n* = 32/36) and 8% (*n* = 3/36) of patients had died from infections. Median time from transplantation to relapse was 9.0 months (range: 0.4–118 months). Median post-relapse OS in 36 relapsed patients was 6.4 months (95% CI: 3.5–9.1 months) (Figure S1C). Six out of the 36 patients that relapsed showed late relapse (3 years post allo-HSCT), including 3 patients with very late relapse that occurred after 8, 9, and 10 years, all of which were located in the BM only.

**Fig. 2:**
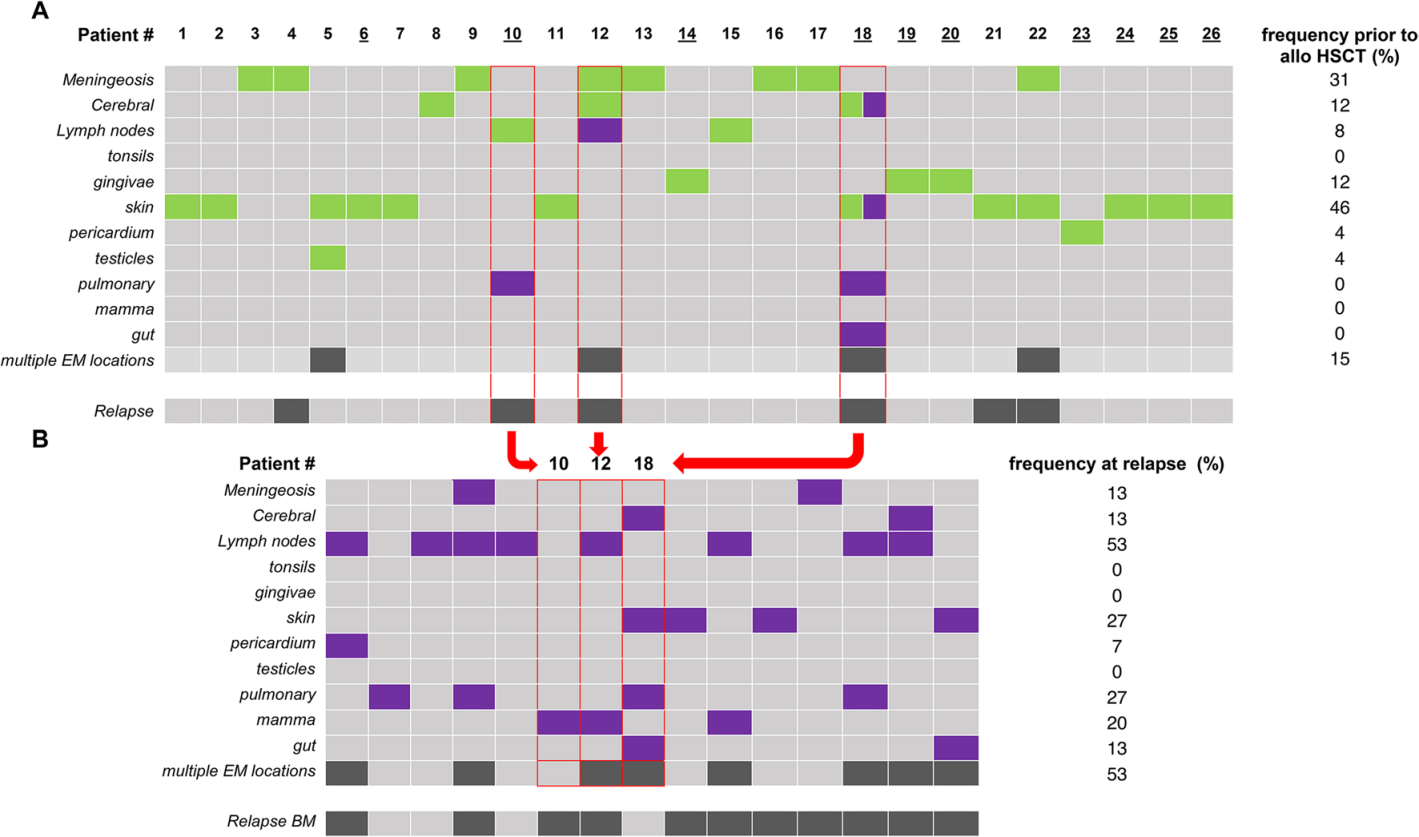
Organ involvement in EM AML. (**A**) Extramedullary manifestations in 26 patients with EMD prior to allo HSCT. (**B**) Extramedullary manifestations in 15 patients with EM ± BM relapse after allo HSCT. Green boxes indicate manifestations prior to allo HSCT. Purple squares represent relapse manifestations. Underlined patients presented with EM AML at first diagnosis (*n* = 10)

While relapse rates were not different between the EMD and non-EMD group, regarding the relapse manifestation site, only three patients (patient numbers #10, #12, #18) with prior EM AML showed EM ± BM relapse, affecting new organ sites in all but one case (Fig. 2A and B). The other 12 patients presenting with EM ± BM relapse had no history of EMD (Fig. 2B).

EMD manifestations after allo-HSCT occurred more often at multiple sites compared to EMD manifestations before allo-HSCT or at initial diagnosis (53% vs 15%, respectively), and showed a different organ site distribution (Fig. 2A and B).

EM manifestation at first diagnosis corresponded to dispersed tissue infiltration by blast cells without formation of a tumor mass. Predominantly, the skin (50%) and gingiva (40%) were involved while hardly ever the CNS (10%) was included. However, prior to allo-HSCT meningeosis leucemica (31%) and CNS involvement (12%) were among the most frequent sites affected (Fig. 2A). Patients with EM involvement at relapse post allo-HSCT most commonly (93%) presented with a tumor mass corresponding to a myelosaroma. Mostly lymph nodes were affected (53%), and chloromatous tumor mass infiltration of multiple organs and tissues were observed, histologically proven in 80% of the cases (Fig. 2B).

### Isolated BM and EM ± BM relapse: characteristics and risk factors

Patients with EM ± BM relapse vs isolated BM relapse showed a higher rate of de novo AML (80% vs 52%, *p* = 0.089) and the presence of *NPM1* mutations in CN-AML (100% vs 13%, *p* = 0.058) (Table S1). All patients with isolated BM relapse had received a fully HLA-matched transplant compared to 80% in the combined EM ± BM group, where three patients were transplanted from an HLA-mismatched donor (HLA-match 9/10) (*p* 0.032) (Table S2). Most of the patients underwent transplantation with active disease. Herein the distribution within the relapse groups was similar (*p* = 0.631) (Table S2).

Post-transplant characteristics are depicted in Tables S3. Grade 1 aGVHD occurred in 75% of patients with isolated BM relapse vs 40% of patients with EM ± BM relapse (*p* = 0.096, Table S3), as also aGVHD occurred pre-dominantly in the skin-only in patients with isolated BM (92%) compared to 70% of patients with combined relapse (Table S3). Time until engraftment was similar in both groups of relapses (isolated BM: median 19 days EM ± BM: median 19 days), as well as time to onset of aGvHD (isolated BM: median 17 days EM ± BM:13 days). Time to relapse and time to death were not different between the different relapse types (Table S3).

Risk factors (*p* < 0.100) for the development of an isolated BM relapse included adverse cytogenetic risk according to MRC, an inadequate blast clearance (≥ 10%) at d16 during induction chemotherapy, and relapsed/refractory disease before allogeneic HSCT (Table 2, Tables S4 and S5). Protective factors that reduced the likelihood of isolated BM relapse included de novo AML vs non-de novo AML, the presence of CN-AML, presence of an *NPM1* mutation, development of a grade 2–4 aGVHD as well as presence of cGVHD (Table 2, Tables S4 and S6).

**Table 2:**
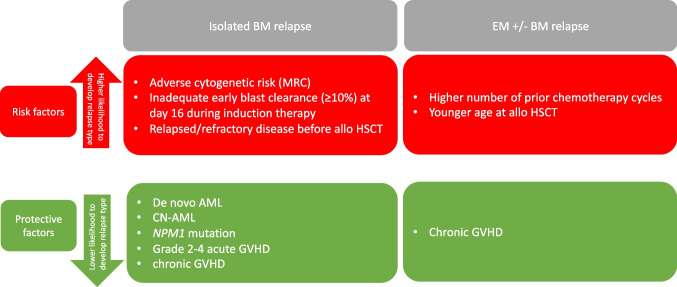
Synoptical table depicting risk factors and protective factors for the development of isolated BM relapse or EM ± BM relapse

This table depicts all pre-transplant (baseline clinical, disease-related), transplant-related (prior therapy, transplant) and post-transplant characteristics with a *p* < 0.01 in a univariate logistic regression model (for further details see Tables S4, S5 and S6)

In contrast, the only risk factors for the development of EM ± BM relapse included younger age at HSCT and a higher number of prior intensive chemotherapy cycles (Table 2, Tables S4 and S5). cGVHD was a protective factor (Table S6).

### Outcome: survival and relapse comparing patients with isolated BM vs EM ± BM relapse

**Fig. 3:**
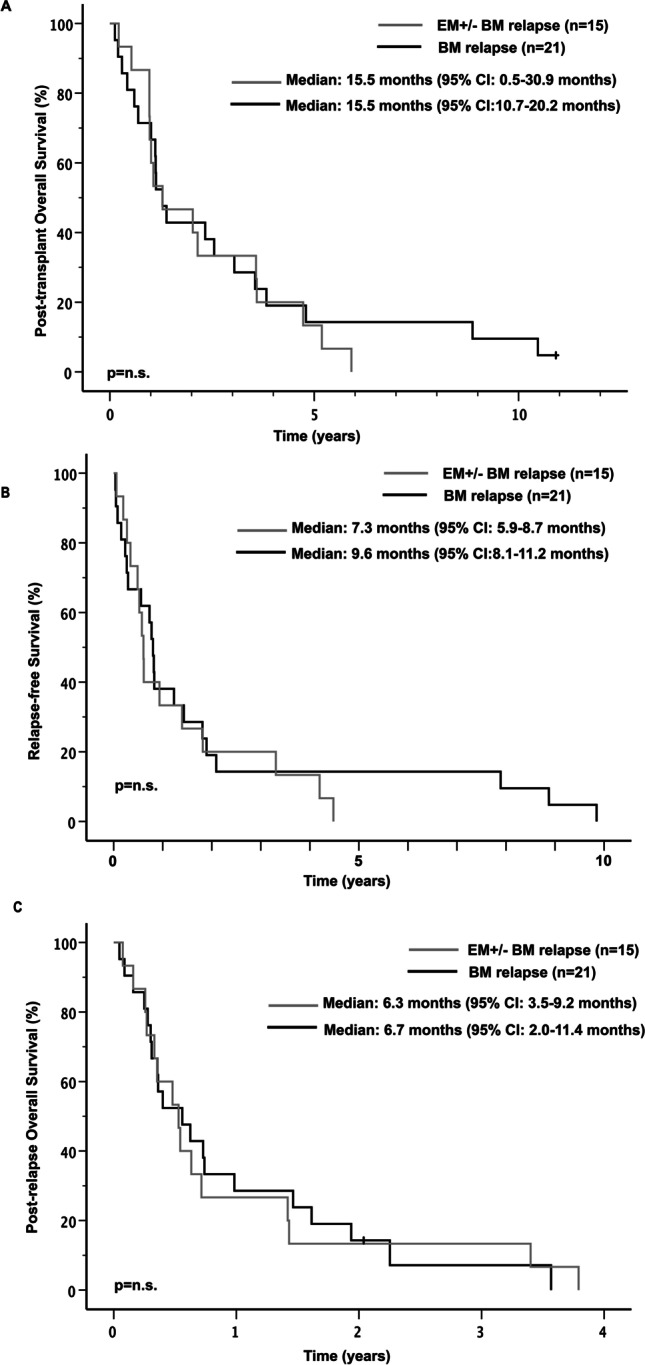
Post-transplant OS (**A**), RFS (**B**) and Post-relapse OS (**C**) in patients with isolated BM versus EM ± BM relapse

Median post-transplant OS in patients with isolated BM relapse and EM ± BM relapse was 15.5 (95% CI: 10.7–20.2) and 15.5 (95% CI: 0.5–30.9) months, respectively, and as such did not reveal significant differences (Fig. 3A). Median post-transplant OS in patients with isolated BM relapse, isolated EM AML relapse and EM + BM relapse was 15.5 (95% CI: 10.7–20.2), 12.1 (95% CI: 0.0–31.1), and 15.5 (95% CI: 1.9–29.1) months, respectively, and did also not reveal significant differences (Figure S2A).

Median RFS was not significantly different in patients with isolated BM relapse compared to patients with EM ± BM relapse (9.6 months; 95% CI: 8.1–11.2 vs. 7.3 months; 95% CI: 5.9–8.7), (Fig. 3B). Median RFS was similar between patients with isolated BM vs. EM ± BM relapse and tended to be longest in patients with isolated EM relapse (Figure S2B).

Median post-relapse OS was similar comparing patients with isolated BM relapse to patients with EM AML relapse with or without concomitant BM relapse (6.7 months; 95% CI: 2.0–11.4 vs. 6.3 months; 95% CI: 3.5–9.2) (Fig. 3C). Median post-relapse OS in patients with isolated BM relapse, isolated EM relapse and EM + BM relapse was 6.7 (95% CI: 2.0–11.4), 3.1 (95% CI: 0.1–6.1) and 7.6 (95% CI: 4.5–10.6) months, respectively (Figure S2C). Patients with isolated EM relapse showed a significant shorter post-relapse OS compared to patients with isolated BM relapse (*p* = 0.049) as well as compared to patients with combined EM + BM relapse (Median post-relapse OS: 3.1 months vs. 7.6 months *p* = 0.014) (Figure S2C). Forty-three percent of patients with isolated BM relapse and 47% of patients with EM ± BM relapse received a second allo-HSCT (data not shown). Only one of the four patients with isolated EM relapse was referred to a potentially curative second allo-HSCT.

When analyses were restricted to patients that had received a treatment with a curative intent (e.g., a second allogeneic HSCT), there were no significant differences with regard to OS and RFS comparing patients with different relapse subtypes (Figures S3 and S4).

Patients with isolated BM relapse and EM with or without concomitant BM relapse showed similar post-relapse OS of 8.9 and 8.6 months, respectively (Figure S5A). One patient with isolated EM relapse showed a shorter post-relapse OS compared to 9 patients with isolated BM relapse (median post-transplant OS: 4.0 months vs. 8.9 months, *p* = 0.069) as well as compared to 6 patients with combined EM + BM relapse (4.0 months vs 8.6 months, *p* = 0.014) (Figure S5B).

## Discussion

The goal of this study was to investigate the occurrence, the prognostic impact, and risk factors for EM AML relapse as well as the association of prior EM AML with outcomes after allogeneic transplantation in a uniformly treated patient cohort receiving sequential FLAMSA-RIC. In the light of limited published data regarding relapse behavior and impact on survival once disease recurred after transplantation, we particularly sought to assess relapse patterns and to identify risk factors for the development of extramedullary relapse after transplantation.

Our study is based on high-risk AML patients receiving a homogeneous conditioning regimen in preparation for HLA-matched transplantation. In our cohort 18% of the patients presented with EMD before transplant. In line with previous publications, regarding clinical presentation at first diagnosis, AML with EM was associated with significantly higher WBC at diagnosis and more frequently tended to harbor *NPM1* and *FLT3*-TKD mutation [30]. Intriguingly, at first diagnosis CNS involvement was rare, however prior to allo-HSCT it made up 43% of EM sites in our cohort. This might be explained by the fact, that we perform CNS staging routinely prior to advancing to allo-HSCT, whereas we do not assess CNS involvement a first diagnosis if the patient presents without related symptoms.

Once transplanted overall relapse rate was 25%, which is similar to data published by Schmid et al [5] (20% relapse rate) in patients with high-risk AML who received FLAMSA-RIC allo-HSCT, but lower compared to other studies in AML patients with various other RIC regimen (30–60% relapse rate) [31, 33]. Rate of EM relapse in our study with 10.4% was moderate in the setting of HLA-matched transplantation. This is similar to 12.9% of EM relapse after RIC for allo-HSCT in high risk AML patients undergoing HLA-matched transplantation reported by Schmid et al. [24], but higher compared to 6–7% in other RIC studies, including all or the majority of patients transplanted in CR [14, 34].

Among the twenty-six patients within our cohort with EMD at diagnosis and prior to transplantation, only three relapsed with EM involvement, underlining the effectiveness of allo-HSCT as a curative treatment also in EM AML. Interestingly, sites affected in patients with EM relapse were multiple showing mostly a solid tumor mass manifestation and mostly did not overlap with the ones at first diagnosis, strongly suggesting that immune escape mechanisms might lead to a different relapse pattern after allo-HSCT [35].

Our study has a long median follow-up time for survivors of 11.6 years, which allows for the detection of late relapses. In fact, 6/36 patients relapsed 3 years after allogeneic HSCT, and another 3 patients developed very late relapses after 8, 9, and 10 years. In contrast to data from Watts et al. who suggested that very late relapses occur predominantly in an EM localization [36] all 3 very late relapses in our study occurred in the BM. However, as at the beginning of the millennium only few mutations were tested in AML patients, it remains unclear whether these late relapses in our cohort were relapses indeed, or on the contrary new therapy-derived AMLs (t-AML) or donor cell derived AMLs as they might nowadays be diagnosed.

Interestingly, risk factors for BM relapses versus EM relapses differed suggesting a difference in their etiology. In addition to known risk factors for the development of relapse such as AML with an adverse cytogenetic risk according to MRC or relapsed/refractory disease before allo-HSCT [3, 17] our study suggests that an inadequate blast clearance (≥ 10%) at d16 during induction therapy is not only a prognostic factor for long-term outcome in AML during conventional chemotherapy [29] but also an indicator of a higher likelihood of BM relapse in the context of allogeneic transplantation. Similarly, the presence of an *NPM1* mutation at diagnosis was found to be a protective factor reducing the likelihood for BM relapse. Rollig et al. showed a longer RFS in patients with *NPM1* + mutated AML after allo-HSCT [37] compared to standard chemotherapy. In this context, a limitation of our study is the lack of MRD data since, e.g., *NPM1*-MRD status post-HSCT has been shown to improve risk assessment for relapse [38]. The only 2 risk factors for the development of EM relapse included younger age and a higher number of induction cycles before allogeneic transplant. Age < 18 years was a risk factor for EM relapse identified by Harris et al. [17]. A higher number of chemotherapy cycles has not been reported before as a risk factor for EM disease but the requirement for more therapy cycles might be a surrogate for a more aggressive disease. Risk factors as disease phase (more relapse/refractory disease) at transplant [17, 21] and poor cytogenetics [17, 19] have been described in other studies that focused on isolated EM disease, in contrast to our analyses that was limited by small numbers of isolated EM relapse and therefore had to combine EM ± BM relapse. While some authors have suggested cGVHD [13, 17] to be associated with EM relapse, this was not observed in a large study performed by Shem-Tov who found that presence of aGVHD or cGVHD was a protective factor for risk of BM relapse, but had no effect on isolated EM relapse risk [19]. In line with these data, we found that aGVHD grade 2–4 lowers the risk for a BM relapse, while it did not affect the risk of EM ± BM relapse. The presence of cGVHD was a protective factor for both types of relapses, reducing the risk of BM as well as of EM ± BM relapse.

Several studies suggest that isolated EM relapse occur later compared to BM relapses [14–16, 19]. We similarly observed a trend to a longer RFS in patients with isolated EM relapse compared to isolated BM relapse. Due to the small number of patients and different treatment strategies post relapse this did not reach statistical significance.

Existing data for prognosis of patients with EM AML relapse compared to BM relapse after allogeneic HSCT is limited and conflicting due to small patient numbers and heterogeneity of patient cohorts and the discrepancy between histologically confirmed and clinically diagnosed EMD. In 80% of our cohort EM AML relapse was histologically confirmed. Shem-Tov et al. demonstrated a better survival of 31 patients with AML or ALL with isolated EM relapses compared to BM relapse. [19]. Similarly, Sohl et al. whose analyses were based on AML patients only found a significantly better post-relapse survival in patients with isolated EM relapses (*n* = 13) compared to those with concurrent BM (*n* = 12) [14]. Chong et al. whose analyses were based on a variety of different hematological malignancies that underwent allo-HSCT including AML, 15 patients with EM ± BM relapse had a favorable post-relapse survival compared to BM relapses [13]. In contrast, one of largest studies including 38 isolated EM relapses and 149 BM relapses in patients with AML or ALL and with a long term follow-up, did not demonstrate differences in outcome depending on the type of relapse [21]. Similarly, Curley et al. did not observe difference in outcome between EM ± BM vs BM relapses [16]. In line with these studies, we did not find a survival benefit for patients with EM ± BM relapse compared to BM relapse. Despite other studies, subgroup analyses differentiating effects of isolated EM from other forms of relapses were challenging due to the small number of patients with isolated EM AML relapse affecting only 4 patients in our cohort. Post-relapse survival tended to be shorted in our four patients with isolated EM relapse, but this was likely due to the fact that 3 of 4 patients had not received another therapy in curative intent and thus did not undergo a potentially curative second allo-HSCT.

Taken together, we observed a moderate occurrence of EM relapses in high-risk AML patients with a feature change to more solid presentation of EMD after FLAMSA-RIC HLA-matched allo-HSCT. We have assessed a wide range of clinical, disease specific, therapeutic and post-transplant characteristics with regard to their impact on BM or EM ± BM relapse risk. Although risk factors for BM vs. EM relapse differed, suggesting a different etiology of these relapse types, overall survival and post-relapse survival was comparable between the two groups. Our long-term follow up enabled us to detect three very late relapses that occurred after 8, 9, and 10 years which emphasizes the need for a long-term close follow-up of AML patients after transplantation. The presence of EMD prior to allo-HSCT did not influence outcomes following sequential-RIC transplantation underlining the effectiveness of this strategy as a curative treatment option also in the treatment of patients with EM AML.

## Supplementary information

Below is the link to the electronic supplementary material.

Supplementary file1 (DOCX 879 KB)
