# Supplementary material for: Impact of extramedullary disease in AML patients undergoing sequential RIC for HLA-matched transplantation: occurrence, risk factors, relapse patterns, and outcome
Source: Ann Hematol. 2023 Jun 10;102(8):2213–23. doi: 10.1007/s00277-023-05281-8 (PMC10344990; doi:10.1007/s00277-023-05281-8)
Supplement: Supplementary file 1 — Supplementary file1 (DOCX 879 KB) [file 277_2023_5281_MOESM1_ESM.docx]

**Impact of Extramedullary Disease in AML patients undergoing sequential RIC for HLA-matched transplantation: Occurrence, Risk factors, Relapse patterns and Outcome**

Supplementary Appendix

| **Table S1: Baseline clinical and disease biology characteristics within 2 different relapse groups** | | | | | | | | | |
| --- | --- | --- | --- | --- | --- | --- | --- | --- | --- |
|  | **Isolated BM relapse (n=21)** | | | | **EM +/- BM relapse (n=15)** | | | | ***P*** |
|  | n | % | median | range | number | % | median | range |  |
| **Clinical characteristics** |  |  |  |  |  |  |  |  |  |
| Age at allo HSCT, years |  |  | 45.1 | 27.0-70.5 |  |  | 43.6 | 21.6-67.1 | 0.170 |
| Female | 9 | 42.9 |  |  | 8 | 53.3 |  |  | 0.535 |
| ECOG at allo HSCT |  |  |  |  |  |  |  |  | 0.588 |
| ECOG 0/1 | 17 | 81.0 |  |  | 11 | 73.3 |  |  |  |
| ECOG 2 | 4 | 19.0 |  |  | 4 | 26.7 |  |  |  |
| Prior EM manifestation at diagnosis | 0 | 0.0 |  |  | 2 | 13.3 |  |  | **0.085** |
| Prior EM manifestation before allo HSCT | 3 | 14.3 |  |  | 3 | 20.0 |  |  | 0.650 |
| HCT-CI score, ≥3 | 3 | 14.3 |  |  | 3 | 20.0 |  |  | 0.650 |
| WBC at diagnosis, x 10^9^/L |  |  | 3.6 | 0.2-252 |  |  | 7.4 | 1.6-232.0 | 0.875 |
| Bone marrow blasts at diagnosis, % |  |  | 42 | 8-93 |  |  | 65 | 30-84 | 0.322 |
| Peripheral blasts at diagnosis, % |  |  | 31 | 1-96 |  |  | 18 | 0-34 | 0.118 |
| **Disease biology** |  |  |  |  |  |  |  |  |  |
| De novo AML | 11 | 52.4 |  |  | 12 | 80.0 |  |  | **0.089** |
| FAB type |  |  |  |  |  |  |  |  | 0.608 |
| M0 | 2 | 12.5 |  |  | 1 | 7.7 |  |  |  |
| M1 | 3 | 18.8 |  |  | 3 | 23.1 |  |  |  |
| M2 | 6 | 37.5 |  |  | 4 | 30.8 |  |  |  |
| M4 | 4 | 25.0 |  |  | 3 | 23.1 |  |  |  |
| M5 | 0 | 0.0 |  |  | 2 | 15.4 |  |  |  |
| M6 | 1 | 6.3 |  |  | 0 | 0.0 |  |  |  |
| M7 | 0 | 0.0 |  |  | 0 | 0.0 |  |  |  |
| Cytogenetic risk group (MRC) |  |  |  |  |  |  |  |  | 0.193 |
| favorable | 0 | 0.0 |  |  | 2 | 14.3 |  |  |  |
| intermediate | 11 | 52.4 |  |  | 7 | 50.0 |  |  |  |
| adverse | 10 | 47.6 |  |  | 5 | 35.7 |  |  |  |
| CN-AML | 7 | 33.3 |  |  | 5 | 33.3 |  |  | 1.000 |
| *NPM1* mutated | 2 | 12.5 |  |  | 3 | 37.5 |  |  | 0.155 |
| *FLT3*-ITD | 4 | 23.5 |  |  | 3 | 30.0 |  |  | 0.711 |
| *FLT3*-TKD | 2 | 22.2 |  |  | 0 | 0.0 |  |  | 0.371 |
| *MLL*-PTD | 1 | 8.3 |  |  | 0 | 0.0 |  |  | 0.402 |
| *NPM1* mutated in CN-AML only | 2 | 12.5 |  |  | 3 | 100.0 |  |  | **0.058** |
| *FLT3*-ITD in CN-AML only | 4 | 23.5 |  |  | 3 | 75.0 |  |  | 0.137 |
| *FLT3*-TKD in CN-AML only | 2 | 22.2 |  |  | 0 | 0.0 |  |  | NA |
| *MLL*-PTD in CN-AML only | 1 | 8.3 |  |  | 0 | 0.0 |  |  | NA |
| Expression of T cell markers before allo HSCT | 10 | 62.3 |  |  | 6 | 50.0 |  |  | 0.508 |
| Expression of CD56 before alloHSCT | 2 | 12.5 |  |  | 3 | 27.3 |  |  | 0.332 |

Bold typing indicates statistical significance (p < 0.1)

| **Table S2: Prior therapy and transplant characteristic within 2 different relapse groups** | | | | | | | | | |
| --- | --- | --- | --- | --- | --- | --- | --- | --- | --- |
|  | **Isolated BM relapse (n=21)** | | | | **EM +/- BM relapse (n=15)** | | | | ***P*** |
|  | number | % | median | range | number | % | median | range |  |
| **Therapy prior to allogeneic HSCT** |  |  |  |  |  |  |  |  |  |
| Prior intensive chemotherapy cycles, number |  |  | 2 | 1-3 |  |  | 2 | 2-4 | 0.281 |
| Prior intensive chemotherapy cycles ≥2 | 3 | 17.6 |  |  | 4 | 30.8 |  |  | 0.400 |
| d16 Blast clearance <10% | 9 | 52.9 |  |  | 7 | 53.8 |  |  | 0.961 |
| Remission status before allo HSCT |  |  |  |  |  |  |  |  | 0.631 |
| CR/CRi | 3 | 14.3 |  |  | 4 | 26.7 |  |  |  |
| Relapse/Refractory | 14 | 66.7 |  |  | 9 | 60.0 |  |  |  |
| Upfront allo HSCT | 4 | 19.0 |  |  | 2 | 13.3 |  |  |  |
| Time from diagnosis to allo HSCT (months) |  |  | 4.4 | 1.3-29.1 |  |  | 6.8 | 1.4-23.2 | 0.340 |
| **Allogeneic HSCT** |  |  |  |  |  |  |  |  |  |
| HLA identical | 21 | 100.0 |  |  | 12 | 80.0 |  |  | **0.032** |
| HLA mismatch | 0 | 0.0 |  |  | 3 | 20.0 |  |  | NA |
| HLA A | 0 | 0.0 |  |  | 1 | 33.3 |  |  |  |
| HLA B | 0 | 0.0 |  |  | 0 | 0.0 |  |  |  |
| HLA C | 0 | 0.0 |  |  | 2 | 66.6 |  |  |  |
| HLA DR | 0 | 0.0 |  |  | 0 | 0.0 |  |  |  |
| HLA DQ | 0 | 0.0 |  |  | 0 | 0.0 |  |  |  |
| Donor type |  |  |  |  |  |  |  |  | **0.075** |
| Related, identical | 10 | 47.6 |  |  | 4 | 26.7 |  |  | 0.204 |
| Unrelated, identical | 11 | 52.4 |  |  | 8 | 53.3 |  |  | 0.955 |
| Unrelated, different | 0 | 0.0 |  |  | 3 | 20.0 |  |  | **0.032** |
| Gender difference | 9 | 42.9 |  |  | 7 | 46.7 |  |  | 0.898 |
| Gender of donor/recipient |  |  |  |  |  |  |  |  | 0.895 |
| Female/female | 4 | 19.0 |  |  | 4 | 26.7 |  |  |  |
| Female/Male | 4 | 19.0 |  |  | 3 | 20.0 |  |  |  |
| Male/Female | 5 | 23.8 |  |  | 4 | 26.7 |  |  |  |
| Male/Male | 8 | 38.1 |  |  | 4 | 26.7 |  |  |  |
| CMV State, donor/recipient |  |  |  |  |  |  |  |  | 0.230 |
| Negative/negative | 7 | 33.3 |  |  | 5 | 33.3 |  |  |  |
| Negative/positive | 0 | 0.0 |  |  | 2 | 13.3 |  |  |  |
| Positive/negative | 7 | 33.3 |  |  | 2 | 13.3 |  |  |  |
| Positive/positive | 7 | 33.3 |  |  | 6 | 40.0 |  |  |  |
| Stem cell source |  |  |  |  |  |  |  |  | NA |
| Peripheral blood | 21 | 100.0 |  |  | 15 | 100.0 |  |  |  |
| Bone marrow | 0 | 0.0 |  |  | 0 | 0.0 |  |  |  |
| Stem cell dose, CD34+cells/kg |  |  | 7.5 | 2.7-18.8 |  |  | 8.6 | 4.8-15.8 | 0.374 |
| Conditioning regime |  |  |  |  |  |  |  |  | 0.669 |
| TBI based | 14 | 66.7 |  |  | 11 | 73.3 |  |  |  |
| Busulfan based | 7 | 33.3 |  |  | 4 | 26.7 |  |  |  |
| TBI based group |  |  |  |  |  |  |  |  | 0.250 |
| FLAMSA- RIC- TBI + ATG 10 mg + Cy | 7 | 50.0 |  |  | 3 | 27.3 |  |  |  |
| FLAMSA- RIC- TBI + ATG 20 mg + Cy | 7 | 50.0 |  |  | 8 | 72.7 |  |  |  |
| Busulfan based group |  |  |  |  |  |  |  |  | 0.898 |
| FLAMSA-RIC-other + ATG 10 mg + Busulfan + Cy | 2 | 28.6 |  |  | 1 | 25.0 |  |  |  |
| FLAMSA-RIC-other + ATG 20 mg + Busulfan + Cy | 5 | 71.4 |  |  | 3 | 75.0 |  |  |  |
| Others | 0 | 0.0 |  |  | 0 | 0.0 |  |  |  |
| GvHD propyhlaxis |  |  |  |  |  |  |  |  | 0.290 |
| CsA / MMF | 17 | 81.0 |  |  | 14 | 93.3 |  |  |  |
| others (Tacrolimus/MMF, Sirolimus/MMF, MTX) | 4 | 19.0 |  |  | 1 | 6.7 |  |  |  |
| Treatment with 2^nd^ HSCT at relapse | 9 | 42.9 |  |  | 7 | 46.7 |  |  | 0.821 |

Bold typing indicates statistical significance (p < 0.1)

| **Table S3: Post-transplant characteristics in patients within 2 different relapse groups** | | | | | | | | | |
| --- | --- | --- | --- | --- | --- | --- | --- | --- | --- |
|  | **Isolated BM relapse (n=21)** | | | | **EM +/- BM relapse (n=15)** | | | | ***P*** |
|  | number | % | median | range | number | % | median | range |  |
| Time until engraftment (days) |  |  | 19 | 14-27 |  |  | 19 | 11-33 | 0.610 |
| Time to onset of aGvHD (days) |  |  | 17 | 9-45 |  |  | 12.5 | 8-47 | 0.228 |
| Time allo HSCT to relapse (months) |  |  | 9.6 | 0.4-118.2 |  |  | 7.3 | 0.7-53.8 | 0.924 |
| Time allo HSCT to death (months) |  |  | 14.5 | 1.5-125.7 |  |  | 15.5 | 2.6-70.9 | 0.805 |
| aGvHD | 12 | 57.1 |  |  | 10 | 66.7 |  |  | 0.563 |
| aGVHD, severity grade |  |  |  |  |  |  |  |  | 0.429 |
| grade I | 9 | 75.0 |  |  | 4 | 40.0 |  |  | **0.096** |
| grade II | 1 | 8.3 |  |  | 2 | 20.0 |  |  | 0.427 |
| grade III | 1 | 8.3 |  |  | 2 | 20.0 |  |  | 0.427 |
| grade IV | 1 | 8.3 |  |  | 2 | 20.0 |  |  | 0.427 |
| aGVHD, severity grade |  |  |  |  |  |  |  |  | 0.221 |
| grade I/II | 10 | 83.3 |  |  | 6 | 60.0 |  |  |  |
| grade III/IV | 12 | 16.7 |  |  | 4 | 40.0 |  |  |  |
| aGvHD, localization |  |  |  |  |  |  |  |  | 0.435 |
| 1-organ: skin | 11 | 91.7 |  |  | 7 | 70.0 |  |  |  |
| 1-organ: intestine | 0 | 0.0 |  |  | 1 | 10.0 |  |  |  |
| 2-organs: skin, intestine | 1 | 8.3 |  |  | 1 | 10.0 |  |  |  |
| 2-organs: skin, liver | 0 | 0.0 |  |  | 0 | 0.0 |  |  |  |
| 3-organs: skin, intestine, liver | 0 | 0.0 |  |  | 1 | 10.0 |  |  |  |
| aGvHD, localization |  |  |  |  |  |  |  |  | 0.190 |
| Isolated skin | 11 | 91.7 |  |  | 7 | 70.0 |  |  |  |
| Non-isolated skin | 1 | 8.3 |  |  | 3 | 30.0 |  |  |  |
| cGvHD | 3 | 14.3 |  |  | 2 | 13.3 |  |  | 0.935 |
| cGvHD, severity grade |  |  |  |  |  |  |  |  | 0.171 |
| Limited | 3 | 100.0 |  |  | 1 | 50.0 |  |  |  |
| Extensive | 0 | 0.0 |  |  | 1 | 50.0 |  |  |  |
| cGvHD, localization |  |  |  |  |  |  |  |  | 0.176 |
| 1-organ: skin | 3 | 100.0 |  |  | 0 | 0.0 |  |  |  |
| 1-organ: intestine | 0 | 0.0 |  |  | 0 | 0.0 |  |  |  |
| 2-organs: skin, intestine | 0 | 0.0 |  |  | 0 | 0.0 |  |  |  |
| 2-organs: skin, liver | 0 | 0.0 |  |  | 1 | 50.0 |  |  |  |
| 3-organs: skin, intestine, liver | 0 | 0.0 |  |  | 1 | 50.0 |  |  |  |
| cGvHD, localization |  |  |  |  |  |  |  |  | **0.025** |
| Isolated skin | 3 | 100.0 |  |  | 0 | 0.0 |  |  |  |
| Non-isolated skin | 0 | 0.0 |  |  | 2 | 100.0 |  |  |  |
| Deaths | 20 | 95.2 |  |  | 15 | 100.0 |  |  | 0.391 |
| Causes of death |  |  |  |  |  |  |  |  | 0.473 |
| AML associated (relapse, refractory disease) | 18 | 90.0 |  |  | 14 | 93.3 |  |  |  |
| Not AML associated (multiple causes per patients) | 2 | 10.0 |  |  | 1 | 6.7 |  |  |  |
| GvHD | 0 |  |  |  | 0 |  |  |  |  |
| Organ toxicity | 0 |  |  |  | 0 |  |  |  |  |
| Infection | 2 |  |  |  | 1 |  |  |  |  |
| Secondary Malignancy | 0 |  |  |  | 0 |  |  |  |  |

Bold typing indicates statistical significance (p < 0.1)

| **Table S4: Clinical and AML Risk factors for isolated BM relapse or EM+/-BM relapse (univariate)** | | | | | | | | |
| --- | --- | --- | --- | --- | --- | --- | --- | --- |
|  |  |  | **Isolated BM relapse** | | | **EM +/- BM relapse** | | |
| **variable** | **comparison** | **N** | **OR** | **95% CI** | **p** | **OR** | **95% CI** | **p** |
| **Clinical characteristics** |  |  |  |  |  |  |  |  |
| Age at allo HSCT, years | Continuous, +1 | 144 | 1.003 | 0.969-1.039 | 0.852 | 0.968 | 0.931-1.006 | **0.098** |
| sex | Female vs male | 144 | 0.738 | 0.290-1.877 | 0.523 | 1.197 | 0.410-3.496 | 0.742 |
| ECOG at allo HSCT | 2 vs 0/1 | 144 | 1.373 | 0.414-4.550 | 0.605 | 2.242 | 0.644-7.812 | 0.205 |
| Prior EM manifestation at diagnosis | Yes vs no | 144 | 0.000 | 0.000-NA | 0.999 | 2.327 | 0.446-12.137 | 0.316 |
| Prior EM manifestation before allo HSCT | Yes vs no | 144 | 0.725 | 0.197-2.668 | 0.628 | 1.152 | 0.301-4.414 | 0.836 |
| HCT-CI score | ≥3 vs <3 | 144 | 0.593 | 0.162-2.163 | 0.428 | 0.944 | 0.249-3.587 | 0.933 |
| WBC at diagnosis, x 10^9^/L | Continuous,+1 | 96 | 1.004 | 0.996-1.013 | 0.340 | 1.001 | 0.989-1.013 | 0.884 |
| Bone marrow blasts at diagnosis, % | Continuous,+1 | 91 | 0.983 | 0.961-1.006 | 0.154 | 1.005 | 0.974-1.036 | 0.765 |
| Peripheral blasts at diagnosis, % | Continuous,+1 | 57 | 1.000 | 0.976-1.025 | 0.970 | 0.964 | 0.920-1.011 | 0.134 |
| **Disease biology** |  |  |  |  |  |  |  |  |
| Origin of AML | De novo vs non-de novo | 144 | 0.403 | 0.157-1.037 | **0.060** | 1.798 | 0.481-6.723 | 0.383 |
| FAB type | M4/M5 vs others | 123 | 0.657 | 0.198-2.184 | 0.493 | 1.339 | 0.409-4.390 | 0.630 |
| Cytogenetic risk group (MRC) | Intermediate vs favorable | 106 | 2x10^9^ | 0.000-NA | 0.999 | 0.402 | 0.073-2.207 | 0.294 |
|  | Adverse vs intermediate | 127 | 3.281 | 1.240-8.680 | **0.017** | 2.219 | 0.653-7.548 | 0.202 |
| Cytogenetic risk group (MRC) | Adverse vs  favorable/intermediate | 139 | 3.755 | 1.423-9.905 | **0.008** | 1.925 | 0.597-6.209 | 0.273 |
| CN-AML | CN-AML vs non-CN-AML | 141 | 0.409 | 0.154-1.086 | **0.073** | 0.426 | 0.138-1.319 | 0.139 |
| *NPM1* mutated | Present vs absent | 110 | 0.230 | 0.049-1.072 | **0.061** | 1.149 | 0.259-5.089 | 0.855 |
| *FLT3*-ITD | Present vs absent | 114 | 0.798 | 0.239-2.663 | 0.713 | 1.163 | 0.281-4.814 | 0.835 |
| *FLT3*-TKD | Present vs absent | 64 | 3.643 | 0.560-23.688 | 0.176 | 0.000 | 0.000-NA | 0.999 |
| *MLL*-PTD | Present vs absent | 98 | 0.778 | 0.090-6.747 | 0.820 | 0.000 | 0.000-NA | 0.999 |
| Expression of T cell markers before allo HSCT | Present vs absent | 117 | 1.993 | 0.673-5.899 | 0.213 | 1.100 | 0.333-3.633 | 0.876 |
| Expression of T cell markers at diagnosis | Present vs absent | 64 | 4.298 | 0.995-18.569 | **0.051** | 0.000 | 0.000-NA | 0.998 |
| Expression of CD56 before alloHSCT | Present vs absent | 116 | 0.609 | 0.128-2.909 | 0.534 | 1.812 | 0.438-7.503 | 0.412 |
| Expression of CD56 at diagnosis | Present vs absent | 64 | 0.222 | 0.026-1.893 | 0.169 | 2.529 | 0.329-19.430 | 0.372 |
|  |  |  |  |  |  |  |  |  |
| Bold typing indicates statistical significance (p < 0.1) | | | | | | | | |

| **Table S5: Therapeutic and transplant-related risk factors for isolated BM relapse or EM relapse+/-BM relapse (univariate)** | | | | | | | | |
| --- | --- | --- | --- | --- | --- | --- | --- | --- |
|  |  |  | **Isolated BM relapse** | | | **EM +/- BM relapse** | | |
| **variable** | **comparison** | **N** | **OR** | **95% CI** | **p** | **OR** | **95% CI** | **p** |
| **Therapy prior to allogeneic HSCT** |  |  |  |  |  |  |  |  |
| Prior intensive chemotherapy cycles, number | Continuous, +1 | 130 | 0.964 | 0.392-2.366 | 0.936 | 3.136 | 1.276-7.703 | **0.013** |
| Prior intensive chemotherapy cycles | ≥2 vs <2 | 130 | 1.210 | 0.314-4.665 | 0.782 | 2.806 | 0.772-10.197 | 0.117 |
| d16 Blast clearance, % | ≥10 vs <10 | 130 | 2.698 | 0.950-7.663 | **0.062** | 2.486 | 0.774-7.983 | 0.126 |
| Remission status before allo HSCT | Relapse/Refractory vs CR/CRi | 130 | 4.122 | 1.123-15.134 | **0.033** | 1.800 | 0.525-6.176 | 0.350 |
|  | Upfront vs Relapse/Refractory | 88 | 1.714 | 0.468-6.273 | 0.415 | 1.204 | 0.231-6.276 | 0.826 |
| Remission status before allo HSCT | Upfront/ Relapse/Refractory vs CR/CRi | 144 | 4.543 | 1.272-16.230 | **0.020** | 1.857 | 0.561-6.149 | 0.311 |
| Time from diagnosis to allo HSCT (months) | Continuous, +1 | 144 | 0.957 | 0.896-1.022 | 0.186 | 0.975 | 0.915-1.040 | 0.446 |
| **Allogeneic HSCT** |  |  |  |  |  |  |  |  |
| HLA status donor - recipient | ident vs different | 144 | 3x10^9^ | 0.000-NA | 0.998 | 0.607 | 0.155-2.375 | 0.473 |
| Donor type | Unrelated identical vs related identical | 124 | 0.617 | 0.239-1.589 | 0.317 | 1.246 | 0.354-4.390 | 0.732 |
|  | Unrelated different vs. unrelated identical | 97 | 0.000 | 0.000-NA | 0.998 | 1.522 | 0.365-6.354 | 0.565 |
| Donor type | related vs unrelated | 144 | 0.473 | 0.185-1.210 | 0.118 | 1.375 | 0.414-4.572 | 0.603 |
| Gender difference | Present vs absent | 144 | 0.958 | 0.376-2.440 | 0.929 | 1.141 | 0.390-3.334 | 0.810 |
| CMV status recipient | Positive vs. negative | 144 | 0.598 | 0.226-1.585 | 0.301 | 1.538 | 0.526-4.495 | 0.432 |
| CMV State, donor/recipient | neg/neg vs all others | 144 | 1.037 | 0.388-2.772 | 0.941 | 1.036 | 0.333-3.222 | 0.952 |
| Stem cell source | bone marrow vs. peripheral blood | 144 | 0.000 | 0.000-NA | 0.999 | 0.000 | 0.000-NA | 0.999 |
| Stem cell dose, CD34+cells/kg | Continuous, +1 | 141 | 0.988 | 0.864-2.239 | 0.855 | 1.021 | 0.880-1.185 | 0.787 |
| Conditioning regime | TBI based vs Busulfan based | 144 | 1.237 | 0.465-3.287 | 0.670 | 1.741 | 0.525-5.767 | 0.365 |
| TBI based, ATG dose, mg | 20 mg vs 10 mg | 90 | 0.551 | 0.175-1.737 | 0.309 | 1.722 | 0.424-6.995 | 0.447 |
| Busulfan based, ATG dose, mg | 20 mg vs 10 mg | 50 | 0.486 | 0.078-3.027 | 0.439 | 0.632 | 0.058-6.880 | 0.706 |
| GvHD prophylaxis | CsA / MMF vs others (Tacrolimus/MMF, Sirolimus/MMF, MTX) | 139 | 0.974 | 0.298-3.181 | 0.965 | 3.535 | 0.444-28.176 | 0.233 |
|  | | | | | | | | |
| Bold typing indicates statistical significance (p < 0.1) | | | | | | | | |

| **Table S6: Post-transplant risk factors for isolated BM relapse or EM relapse+/-BM relapse (univariate logistic regression)** | | | | | | | | |
| --- | --- | --- | --- | --- | --- | --- | --- | --- |
|  |  |  | **Isolated BM relapse** | | | **EM +/- BM relapse** | | |
| **variable** |  | N | OR | 95% CI | p | OR | 95% CI | p |
| Time until engraftment (months) | Continuous, +1 | 138 | 3.140 | 0.334-29.517 | 0.317 | 2.071 | 0.157-27.273 | 0.580 |
| Time to onset of aGvHD (months) | Continuous, +1 | 93 | 1.649 | 0.291-9.327 | 0.572 | 0.621 | 0.055-6.978 | 0.700 |
| Time to relapse (months) | Continuous, +1 | 36 | 1.010 | 0.984-1.036 | 0.461 | 0.990 | 0.965-1.016 | 0.461 |
| aGvHD | Present vs absent | 144 | 0.667 | 0.260-1.710 | 0.399 | 1.071 | 0.345-3.327 | 0.905 |
| aGVHD, severity grade | 2-4 vs. 1 | 93 | 0.132 | 0.033-0.532 | **0.004** | 0.764 | 0.199-2.929 | 0.694 |
| cGvHD | Present vs absent | 144 | 0.252 | 0.070-0.900 | **0.034** | 0.243 | 0.053-1.123 | **0.070** |
| cGvHD, severity grade | Extensive vs limited | 52 | 0.000 | 0.000-NA | 0.998 | 1.632 | 0.096-27.648 | 0.735 |
|  |  |  |  |  |  |  |  |  |
| Bold typing indicates statistical significance (p < 0.1) | | | | | | | | |

**Figure S1: Post-transplant overall survival, Relapse-free survival and Post-relapse Overall survival in all patients**

**Figure S2: Post-transplant OS, Relapse-free survival and Post-relapse OS in patients with isolated BM, isolated EM and combined EM and BM relapse**

**Figure S3: Post-transplant OS in patients with different types of relapse treated with curative intent (2^nd^ allogeneic HSCT)**

**Figure S4: Relapse-free survival in patients with different types of relapse treated with curative intent (2^nd^ allogeneic HSCT)**

**Figure S5: Post-relapse OS in patients with different types of relapse treated with curative intent (2^nd^ allogeneic HSCT)**
